# Screening Retinopathy of Prematurity in Extremely Low Birth Weight Infants in China and the Need for Earlier Screening Times

**DOI:** 10.1155/2016/7065835

**Published:** 2016-12-27

**Authors:** Jianxun Wang, Feng Chen, Shiping He, Daoman Xiang

**Affiliations:** Department of Ophthalmology, Guangzhou Women and Children's Medical Center, The Affiliated Hospital of Guangzhou Medical University, Guangzhou 510623, China

## Abstract

*Purpose*. To convey the need for a revised screening strategy for retinopathy of prematurity (ROP) for extremely low birth weight (ELBW) infants in China.* Design*. A retrospective longitudinal study.* Methods*. The medical charts of infants with a birth weight (BW) of less than 1 kg were reviewed. The infants were divided into three groups: group A, without ROP; group B, with ROP but not up to type 1 prethreshold or threshold ROP; group C, with type 1 prethreshold or threshold ROP. Data collected included gender, gestational age (GA), BW, postmenstrual age (PMA), age of onset of ROP, and age at which treatment was carried out, if required.* Results*. A total of 77 infants were involved. Fifty-six infants developed ROP at any stage and 38 infants developed type 1 prethreshold or threshold ROP. The mean BW and GA of infants in group A were significantly different compared with groups B and C. The mean PMA of onset of ROP in infants who developed mild ROP was 37 weeks compared with 34 weeks for infants who developed severe ROP.* Conclusion*. ELBW infants have a higher incidence of ROP in China which highlights the need for optimizing neonatal care for these infants. In ELBW infants, ROP tends to develop more severely when it occurs earlier. It is necessary for ELBW infants, especially for those with a BW less than 800 g or a GA less than 25 weeks, to be initially screened at an earlier time.

## 1. Introduction

Retinopathy of prematurity (ROP) is a major cause of blindness in children both in the developing and in the developed world [1, 2]. Birth weight (BW) and gestational age (GA) are major risk factors for ROP [3]. Extremely low birth weight (ELBW) infants have a higher risk of developing severe ROP and have a higher need for treatment than more mature infants [4]. Timely screening of at-risk infants by an experienced ophthalmologist with a binocular indirect ophthalmoscope or RetCam is important for preventing blindness and effective ROP screening programs have been established in developed countries founded on evidence-based studies [5]. However, in developing countries such as China, there is no evidence-based screening strategy for ROP. Comparison of populations of infants with severe ROP in developed countries with those in developing countries shows a wide range of BW and GA ages for severe ROP [1]. Therefore, many at-risk premature infants do not receive timely screening due to lack of equipment, availability of ophthalmologists, and many other reasons, which may lead to severe visual impairment, especially in those with an extremely low BW. We conducted this retrospective longitudinal study, which focused on the relationship between extremely low BW infants and ROP in a developing country. The data was collected from a local government guided-network for ROP prophylaxis and treatment in Guangzhou, China.

## 2. Materials and Methods

This retrospective longitudinal study was conducted at Guangzhou Women and Children's Medical Center. The study was approval by the Guangzhou Women and Children's Medical Center Review and Ethics Board. The parents of all children involved in the study provided written informed consent to participate. Inclusion criteria for infants in this study were a BW less than 1000 g; a patient at the neonatal intensive care unit; and a completed ROP screening between January 2010 and July 2015. Infants without complete ROP screening outcomes and infants who died prior to ROP screening were excluded. Data were derived from a Guangzhou government guided network for ROP prophylaxis and treatment, which consisted of one central city level department for ROP identification and treatment and 10 affiliated district level departments for ROP primary screening.

Infants were initially screened using an indirect binocular ophthalmoscope at 32 weeks' postmenstrual age (PMA) or four weeks after birth, in accordance with the Chinese Medical Association Guild suggestion. Follow-up examinations were performed every 2 to 14 days until retinal vasculature reached maturity, until any ROP regressed, or until ROP required treatment. Infants were treated in accordance with the “Early Treatment of ROP” recommendations, including those with type 1 and threshold ROP [6]. Any infant who was suspected of requiring treatment in an affiliated district level department was sent to the central city level department for identification or further treatment. Oxygen supplementation of these infants followed the guidelines for oxygen therapy of premature infants in China which indicates incubation at 89%–94% saturated O_2_ [7].

The infants were divided into 3 groups: group A, without any ROP; group B, with ROP but not up to type 1 prethreshold or threshold ROP; group C, with type 1 prethreshold or threshold ROP. Data collected included gender, gestational age (GA), BW, PMA, age of onset of ROP, and age at which treatment was carried out, if required. GA and PMA were defined according to the American Academy of Pediatrics [8]. GA was defined as the time elapsed between the first day of the last menstrual period and birth. PMA was defined as the time elapsed between the first day of the last menstrual period and birth plus the chronologic age. ROP was classified according to the international classification of ROP [9].

Data were statistically analyzed using Statistical Package for the Social Sciences ver.19.0 software (SPSS. Chicago, IL). Probability values of less than 5% were considered to be significant.

## 3. Results

A total of 196 eyes of 98 infants were included in our study. Of the 98 infants, 9 were excluded because they died prior to ROP screening and 12 were excluded because they did not complete a ROP screening. Thirty-eight infants (49%) developed type 1 prethreshold or threshold ROP. None of the infants were diagnosed with stage 4 or stage 5 ROP. Data collected from groups A, B, and C are listed in Table 1. The mean BW for all the included infants was 901.6 ± 109.3 g and the mean GA was 28.2 ± 1.8 weeks. The mean BW of infants who developed any ROP was 898.7 ± 102.6 g and the mean GA was 27.9 ± 1.6 weeks. Furthermore, the mean BW of infants who did not have any ROP was 898.6 ± 102.6 g and the mean GA was 29.1 ± 2.0 weeks. The mean BW of infants who did not have any ROP was not statistically different from those that did (*P* > 0.05); however the mean GA was (*P* < 0.05) listed in Table 2.

In group B, which consisted of infants who developed mild ROP, the mean PMA of onset of ROP was statistically different (*P* < 0.01, two-sided independent-samples Student's *t*-test) compared with group C (Table 1).

For group C, the range of the PMA of treatment was 31 to 42 weeks and the mean PMA was 36.7 ± 2.4 weeks (95% confidence interval 36.0 ± 1.8 to 37.5 ± 2.8). Two infants in group C were diagnosed with zone 1 stage 3 with plus ROP at their first screening at PMA 31 weeks. The BW of these two infants was 670 g and 710 g and GA were 25 weeks for both.

## 4. Discussion

There were three epidemics of ROP in the world since 1950. The “first epidemic” occurred in the 1950s due to unrestricted oxygen supplementation [10, 11]. After oxygen supplementation was controlled, as modern neonatal care improved, many ELBW infants survived. ROP blindness began to reemerge during the second epidemic [11]. Both epidemics mainly occurred in developed countries. In the last decade there was a third epidemic of ROP in middle-income countries [1, 12–14]. The reasons for the latest epidemic are unknown and differ from previous occurrences. For example, there was a variation in the incidence of ROP in infants born with similar GA due to different levels of neonatal care [15] and threshold ROP could be seen in infants born with a BW of 2000 g [16]. However, few reports of the epidemic of ROP in ELBW in developing countries have been published, and these infants have a significant risk of blindness due to ROP.

In China and around the world, the survival rate of extremely premature infants has been increasing as neonatal care improved [17]. Historically, the regional incidence and severity of ROP have been measured in developed countries [18, 19]. In our study, the overall incidence of any stage of ROP was higher than the incidence for the second largest neonatal intensive care unit in Canada [20]. Considering the infants enrolled in the Canadian study were more premature than those in our study, the higher incidence of ROP in our study implicates the need for optimizing neonatal care for ELBW infants in developing countries.

According to several studies [21–24], there is a negative correlation between the incidences of ROP and GA. In our study, the mean GA between infants with any stage of ROP and without any ROP was statistically significant; however, the mean BW between the two groups was not. The possible reason for this outcome may be that the fluctuation range of infants' BW in our study was small leading to statistically insignificant results. Indeed, the BW of the infants in our study was limited to 800–1000 g. For the 98 infants included in our study, only 5 had a BW less than 800 g.

In our study, the mean PMA of onset of ROP was higher, 35 weeks, compared with 34 weeks measured in two large multicenter studies in developed countries [25]. At a PMA of 35 weeks, in China, most premature infants are released from the hospital and face the risk of missed diagnosis, which suggests informing, transforming, and making subsequent screening services available for discharged infants, key points to be considered for improving ROP prophylaxis and treatment.

In this study, the onset of ROP for infants in group C was earlier compared with group B suggesting that the earlier the ROP occurs, the greater the chance of developing severe ROP. The onset time of ROP needed for treatment was 36 weeks [25] according to the ETROP and CRYO-ROP studies, which is similar to our study at 36.7 weeks. Considering the high risk of developing severe ROP and the lack of medical resources in China, guardians of extremely premature infants should be advised to make follow-up arrangements to prevent severe ROP that may have been missed when they were discharged from the hospital.

The American Academy of Pediatrics revised the ROP screening schedule in 2013, suggesting an initial screening time of 31 weeks' PMA or four weeks after birth, whichever is later [5]. The revised statement also suggested infants born before 25 weeks' GA should be considered for earlier screening (six weeks' chronologic age, even if before 31 weeks' PMA) to identify and treat posterior ROP, a particular form of ROP that rapidly progresses to advanced stages and is more likely to occur in ELBW infants. In the United Kingdom, five infants were diagnosed with stage 4 or 5 ROP in an initial screening at six weeks postnatally and included some ELBW infants, with the smallest infant having a BW of 448 g and a GA of 23 weeks [26]. In our study, two infants with a BW of 670 g and 710 g and GA of 25 weeks were diagnosed with threshold ROP (zone 1 stage 3 plus ROP) at their initial screening of PMA 31 weeks. If these two infants were screened earlier, they would have been diagnosed as type 1 prethreshold ROP instead of threshold ROP, which may have led to a better prognosis. Therefore, the current screening strategy should be revised for extremely premature infants.

## 5. Conclusion

ELBW infants have a higher incidence of ROP in China which highlights the need to optimize neonatal care for these infants. Analyzing and transforming screening services are key to improving ROP prophylaxis and treatment, along with availability of services. In ELBW infants, ROP tends to develop more severely when it occurs earlier. It is necessary for ELBW infants, especially for those with a birth weight less than 800 g or a gestational age less than 25 weeks, to be initially screened at an earlier time point.

## Figures and Tables

**Table 1:** Demographics of different group infants with birth weight ≤ 1000 g.

Variables	Group A (no rop) (*n* = 21)	Group B (light) (*n* = 18)	Group C (severe) (*n* = 38)	*P* value
Gender, *n* (%) male	16 (16/21) (76.2%)	11 (11/18) (61.1%)	26 (26/38) (68.4%)	0.597^*※*^
Birth weight (g), mean (SD)	898.6 (102.6)	936.7 (101.5)	886.6 (115.3)	0.277^&^
Gestational age (weeks), mean (SD)	29.1 (2.0)	28.0 (2.0)	27.8 (1.4)	0.016^&^
PMA of onset of ROP (weeks), mean (SD)		36.5 (1.9)	34.3 (1.7)	0.01^&^

^*※*^Chi square test.

^&^Analysis of variance.

SD: standard deviation.

PMA: postmenstrual age.

**Table 2:** Different birth weight and gestational age between infants with or without retinopathy of prematurity.

Variables	Infants without any ROP (*n* = 21)	Infants with any ROP (*n* = 56)	*P* value
Birth weight (g), mean (SD)	898.6 (102.6)	898.7 (102.6)	0.885^&^
Gestational age (weeks), mean (SD)	29.1 (2.0)	27.9 (1.6)	0.004^&^

^&^Analysis of variance.

SD: standard deviation.
